# Supplementary material for: Seasonal change of Burkholderia pseudomallei in paddy field water strongly correlates with ambient temperature: A study in north-central Vietnam
Source: PLoS Negl Trop Dis. 2025 Jul 30;19(7):e0013322. doi: 10.1371/journal.pntd.0013322 (PMC12321097; doi:10.1371/journal.pntd.0013322)
Supplement: S1 Table — (DOCX) [file pntd.0013322.s001.docx]

**S1 Table.** Summary of daily weather data from four weather stations in 2018.

|  | Weather station | Max temp (^o^C) | Min temp (^o^C) | Average temp  (^o^C) | Total precipitation (mm) | Min humidity  (%) | Average humidity  (%) | Max wind speed (m/s) |
| --- | --- | --- | --- | --- | --- | --- | --- | --- |
| Min | Bai Thuong | 13.3 | 9.5 | 11.5 | 0.0 | 36.0 | 64.0 | 0 |
| Mean |  | 28.7 | 21.6 | 24.2 | 6.2 | 66.9 | 86.4 | 0.89 |
| Median |  | 29.8 | 22.9 | 25.5 | 0.0 | 66.0 | 88.0 | 1 |
| Max |  | 41.0 | 29.0 | 34.3 | 120.0 | 95.0 | 98.0 | 5 |
| Min | Nhu Xuan | 13.1 | 8.5 | 11.5 | 0.0 | 32.0 | 60.0 | 0 |
| Mean |  | 28.4 | 21.6 | 24.2 | 5.9 | 67.9 | 86.6 | 1.29 |
| Median |  | 29.2 | 22.9 | 25.2 | 0.0 | 68.0 | 88.0 | 1 |
| Max |  | 39.7 | 30.8 | 34.5 | 116.6 | 95.0 | 98.0 | 7 |
| Min | Thanh Hoa | 13.1 | 9.6 | 11.3 | 0.0 | 34.0 | 52.0 | 1 |
| Mean |  | 28.0 | 22.3 | 24.5 | 4.9 | 67.0 | 83.4 | 2.64 |
| Median |  | 28.5 | 23.5 | 25.6 | 0.0 | 67.0 | 85.0 | 3 |
| Max |  | 40.2 | 30.9 | 34.7 | 117.3 | 93.0 | 97.0 | 7 |
| Min | Yen Dinh | 13.1 | 9.7 | 11.0 | 0.0 | 29.0 | 58.0 | 0 |
| Mean |  | 28.1 | 22.0 | 24.3 | 3.9 | 68.6 | 86.3 | 2.81 |
| Median |  | 28.9 | 23.2 | 25.4 | 0.0 | 69.0 | 88.0 | 3 |
| Max |  | 40.0 | 30.4 | 34.4 | 60.1 | 95.0 | 98.0 | 9 |
| Min | Average for 4 stations | 13.3 | 9.8 | 11.5 | 0.0 | 33.0 | 61.0 | 0.5 |
| Mean |  | 28.3 | 21.9 | 24.3 | 5.2 | 67.6 | 85.7 | 1.91 |
| Median |  | 29.1 | 23.1 | 25.6 | 0.1 | 67.3 | 87.3 | 1.75 |
| Max |  | 40.1 | 30.3 | 34.5 | 83.3 | 93.8 | 96.3 | 6.25 |
