# Supplementary material for: Seasonal change of Burkholderia pseudomallei in paddy field water strongly correlates with ambient temperature: A study in north-central Vietnam
Source: PLoS Negl Trop Dis. 2025 Jul 30;19(7):e0013322. doi: 10.1371/journal.pntd.0013322 (PMC12321097; doi:10.1371/journal.pntd.0013322)
Supplement: S2 Table — (DOCX) [file pntd.0013322.s002.docx]

**S2 Table.** Pearson pairwise correlations between weather variables in 2018.

|  | Max temp | Min temp | Average temp | Total precipitation | Min humidity | Average humidity | Max win speed |
| --- | --- | --- | --- | --- | --- | --- | --- |
| Max temp | 1.000 |  |  |  |  |  |  |
| Min temp | 0.907 | 1.000 |  |  |  |  |  |
| Average temp | 0.969 | 0.978 | 1.000 |  |  |  |  |
| Total precipitation | 0.061 | 0.185 | 0.133 | 1.000 |  |  |  |
| Min humidity | -0.340 | -0.036 | -0.181 | 0.342 | 1.000 |  |  |
| Average humidity | -0.128 | 0.061 | -0.038 | 0.312 | 0.860 | 1.000 |  |
| Max wind speed | -0.024 | -0.052 | -0.033 | 0.188 | -0.046 | -0.162 | 1.000 |
