# Supplementary material for: Seasonal change of Burkholderia pseudomallei in paddy field water strongly correlates with ambient temperature: A study in north-central Vietnam
Source: PLoS Negl Trop Dis. 2025 Jul 30;19(7):e0013322. doi: 10.1371/journal.pntd.0013322 (PMC12321097; doi:10.1371/journal.pntd.0013322)
Supplement: S3 Table — (DOCX) [file pntd.0013322.s003.docx]

**S3 Table.** Summary of demographic characteristics of 40 culture-confirmed melioidosis cases in Nghe An and Ha Tinh provinces in 2018.

|  | **All cases  (n=40)** | **Case cluster 1  (n=8)** | **Case cluster 2  (n=16)** | **Case cluster 3  (n=8)** |
| --- | --- | --- | --- | --- |
| **Median age (IQR; range)** | 57.0y (46.8y-66.8y; 14y-92y) | 58.0y (50.3y-67.3y; 47y-83y) | 57.0y (42.5y-66.0y; 14y-86y) | 50.5y (41.0y-60.5y; 22y-92y) |
| **Male sex** | 31 (78%) | 5 (63%) | 14 (88%) | 7 (88%) |
| **Rural residence** | 38 (95%) | 8 (100%) | 15 (94%) | 7 (88%) |
| **Occupation** |  |  |  |  |
| Student | 1 (2.5%) | - | 1 (6.25%) | - |
| Rice farmer | 13 (32.5%) | 2 (25%) | 6 (37.5%) | 3 (37.5%) |
| Elderly people* | 12 (30%) | 3 (37.5%) | 4 (25%) | 1 (12.5%) |
| Retired officer | 2 (5%) | - | 1 (6.25%) | 1 (12.5%) |
| Unknown | 12 (30%) | 3 (37.5%) | 4 (25%) | 3 (37.5%) |

* Patients who were 65 years or older at the time of data collection in 2018.
