# Supplementary figures and images for: Seasonal change of Burkholderia pseudomallei in paddy field water strongly correlates with ambient temperature: A study in north-central Vietnam
Source: PLoS Negl Trop Dis. 2025 Jul 30;19(7):e0013322. doi: 10.1371/journal.pntd.0013322 (PMC12321097; doi:10.1371/journal.pntd.0013322)

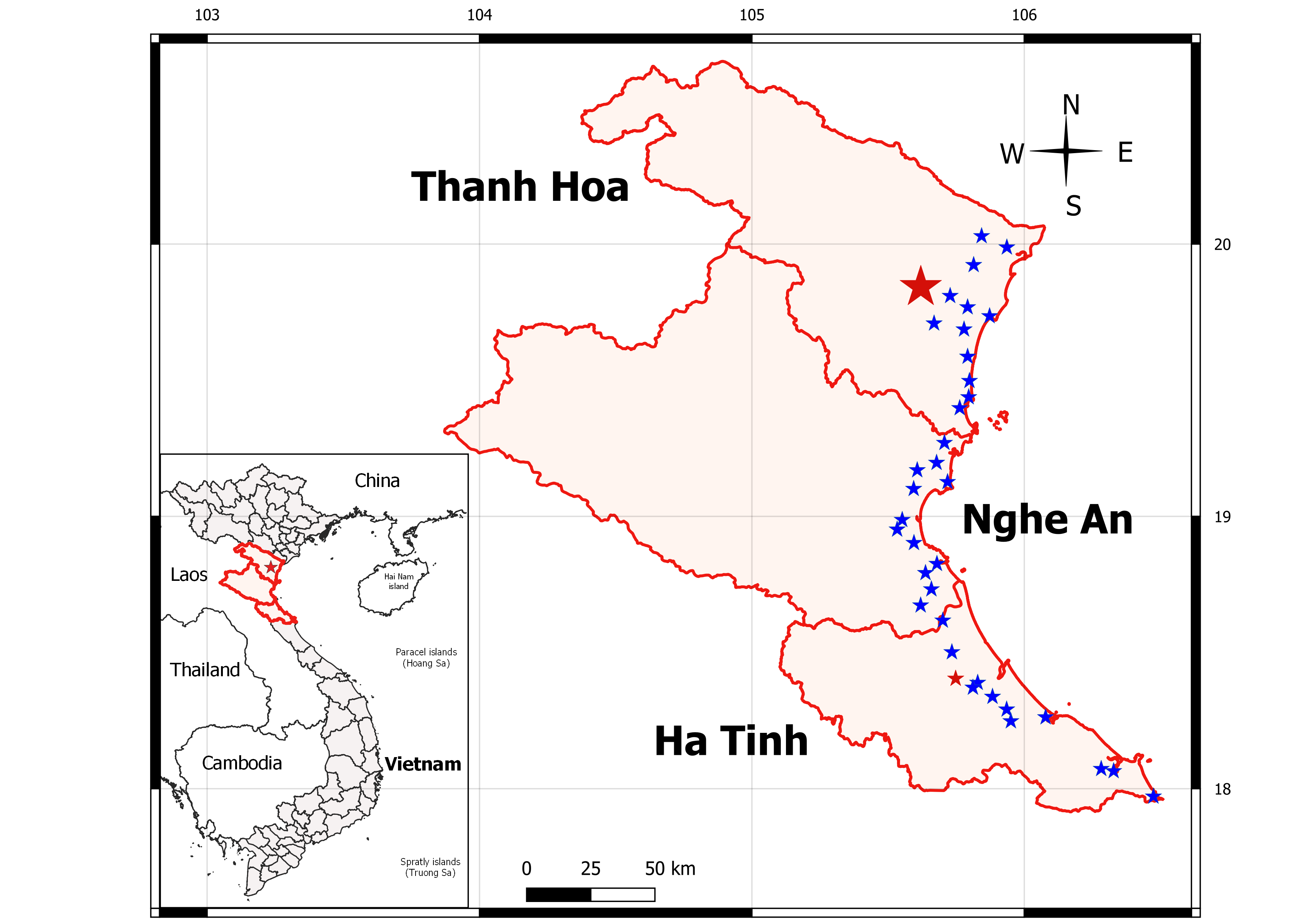

Supplement: S1 Fig — Blue asterisks indicate sampling sites negative for B. pseudomallei, while red asterisks indicate sites positive for B. pseudomallei. Five soil samples were collected at each site, and the size of the asterisks is proportional to the number of B. pseudomallei-positive soil samples per site. The map was created using the QGIS software version 3.22.1. The basemap shapefile was downloaded from the Database of Global Administrative Areas https://gadm.org/download_country.html (TIF) [file pntd.0013322.s004.tif]

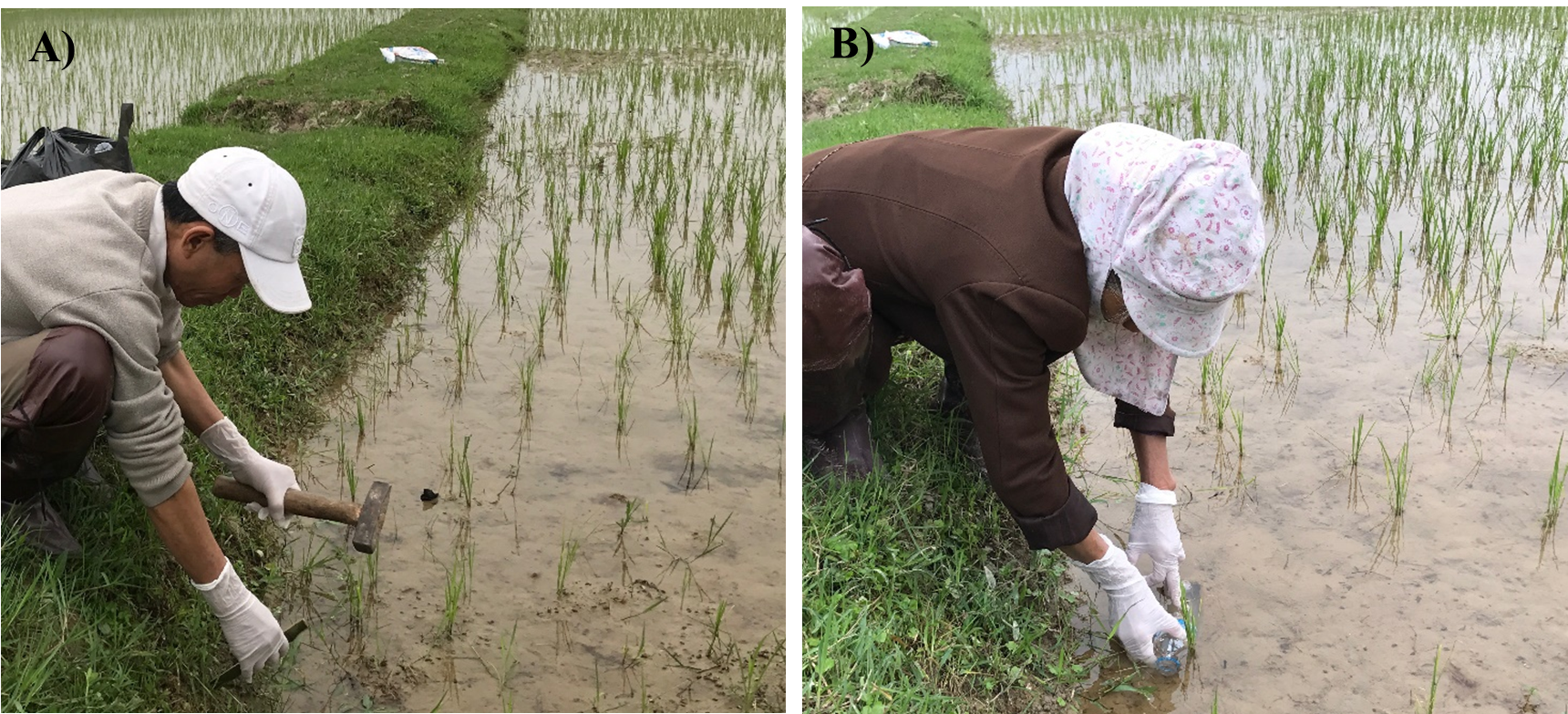

Supplement: S2 Fig — (A) Marking of sampling points with bamboo stakes during the first visit to the rice paddy field in 2018. (B) Sampling of paddy field water using a 500 ml plastic bottle. (TIF) [file pntd.0013322.s005.tif]

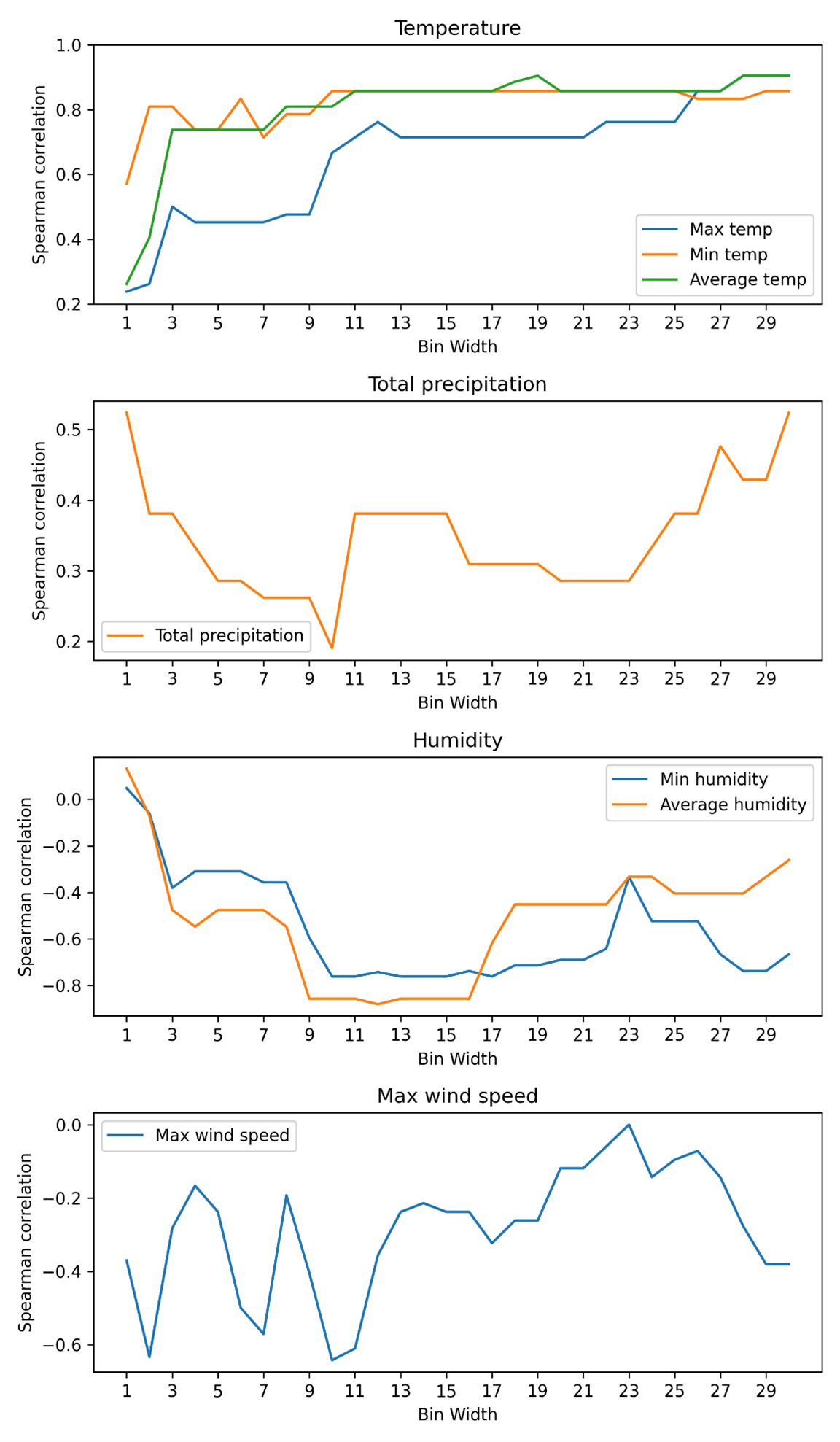

Supplement: S3 Fig — (TIF) [file pntd.0013322.s006.tif]

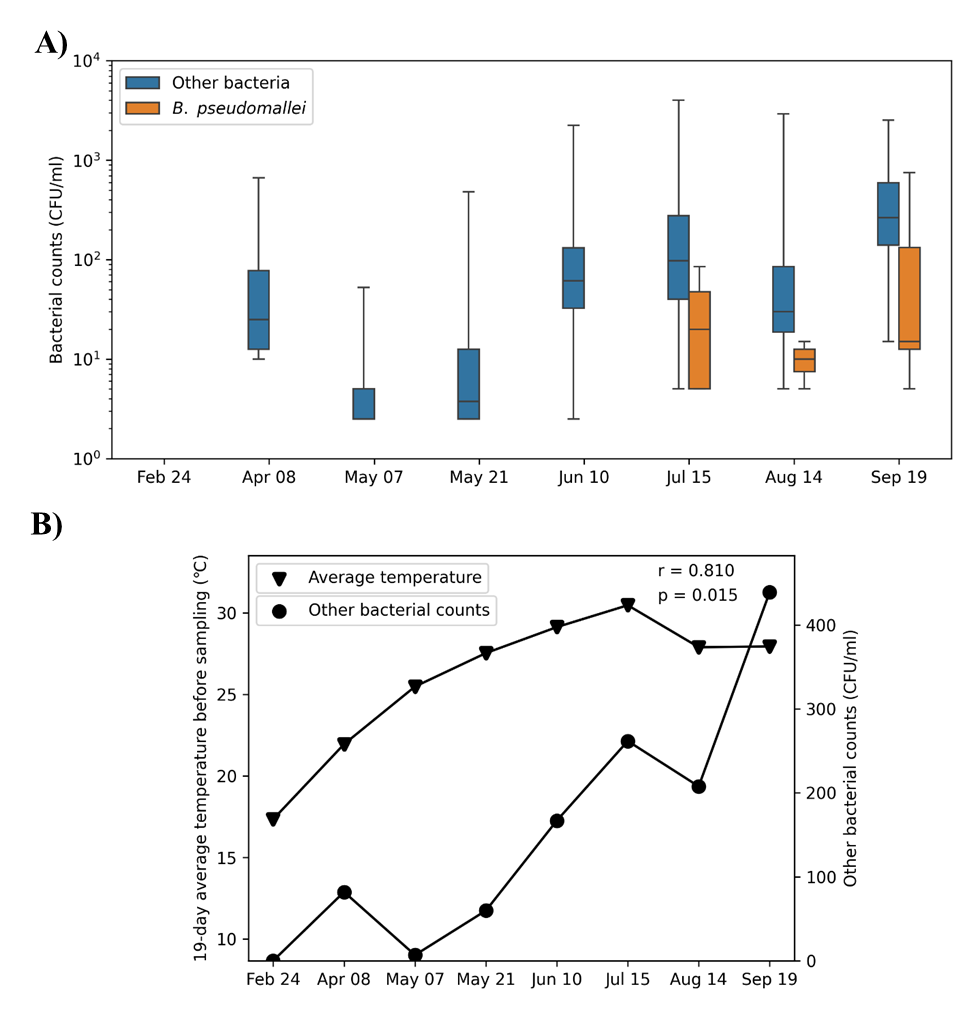

Supplement: S4 Fig — (A) B. pseudomallei and other bacterial counts in the paddy field water over the year 2018. (B) Spearman’s rho correlation between other bacterial counts in the paddy field water and the 19-day bin data of the average ambient temperature. (TIF) [file pntd.0013322.s007.tif]

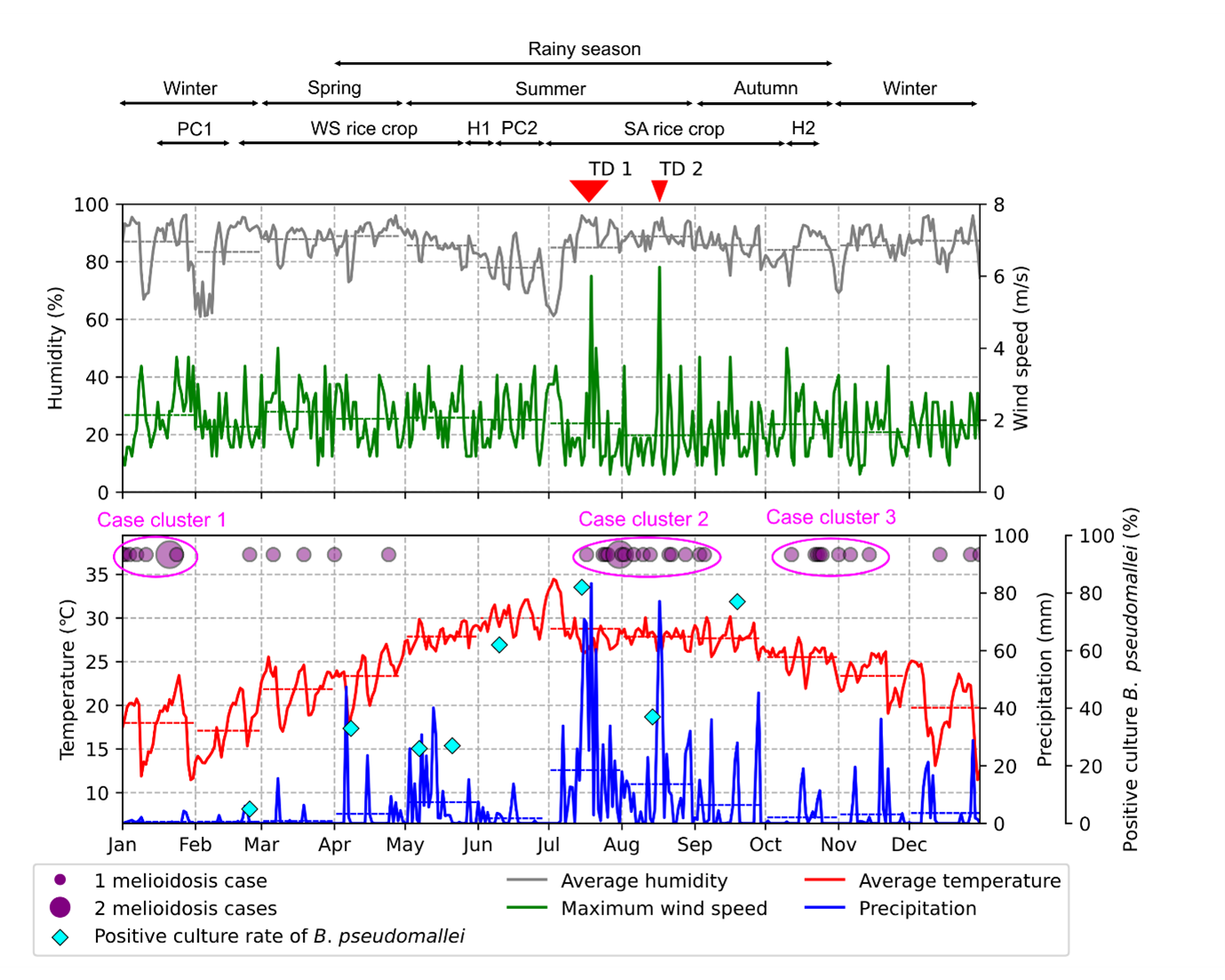

Supplement: S5 Fig — The red, blue, green, and grey lines represent the daily average temperature, total precipitation, maximum wind speed, and average humidity, respectively. The dashed bars show the monthly average weather variables. The cyan diamonds indicate the positive culture rate of B. pseudomallei at different sampling dates. The pink circles represent the dates when melioidosis patients were admitted to the hospitals, with the size of each circle proportional to the number of cases admitted on that day. The pink ovals are clusters of melioidosis cases observed. The red triangles indicate the occurrence of two tropical depressions (TD1 and TD2) that affected north-central Vietnam in 2018. The upper arrows indicate different seasons and the order of agriculture activities throughout the year, including plowing and cultivation (PC1) and harvesting (H1) in the first winter-spring (WS) rice crop, and plowing and cultivation (PC2) and harvesting (H2) in the second winter-spring (WS) rice crop. (TIF) [file pntd.0013322.s008.tif]
