# Supplementary material for: Seasonal change of Burkholderia pseudomallei in paddy field water strongly correlates with ambient temperature: A study in north-central Vietnam
Source: PLoS Negl Trop Dis. 2025 Jul 30;19(7):e0013322. doi: 10.1371/journal.pntd.0013322 (PMC12321097; doi:10.1371/journal.pntd.0013322)
Supplement: Attachment — Submitted filename: Response to Reviewers.docx [file pntd.0013322.s010.docx]

Dear reviewers,

Thank you very much for your thoughtful review and for giving us many valuable comments and suggestions that help us to improve the manuscript. In this letter, we provide our responses to each of your comments below.

Best regards,

Trung

**Reviewers' Comments:**
**Methods**

Reviewer #1: See below

Reviewer #2: Study design appropriate with appropriate methods to address the objectives of the study. Some additional measures could have been included to make findings and conclusions more robust (specifically, water temperature measurements).
Of note, the paragraph lines 223-227 mentions "other bacteria", however the methods related to this aspect are not included in the manuscript; they need to be included in the revision in order to validate the results presented.
Beyond that, sample size and analyses are appropriate and support conclusions.

🡪 Quantification of other bacteria is included in lines 193-203.

Reviewer #3: Overall, the methods used to achieve the objectives of the study were suitable and acceptable. Description of the methods however tended to be on the brief side, requiring reader to search and refer to previous studies. Although this is not unusual, making the effort to describe pertinent experimental details would greatly improve readability and help readers to focus more on the current manuscript. Following are questions and comments that naturally surfaced when the manuscript was reviewed:
Regarding Study Site, L127-131:
i) Although it was mentioned that the current study site was selected because it was the same site on which a pilot study was conducted back in 2015 (line 115), the reasons and justifications for conducting the study on this site were not clear. Might it be that this was a convenient sampling, in which the research team by chance managed to convince the paddy field owner to participate in the study? OR The site was nearby to other sites that were previously reported positive for B. pseudomallei?

🡪 Reason for selecting the study site is included in lines 135-146.
ii) Is the rice field with the coordinate 19deg50’18.8” N;105deg37’13.4” E (that have all 5 soil samples positive for B. pseudomallei) also the same one sampled for water in 2018?

🡪 Yes
iii) The description of the water sampling points in the text although is adequate, it could help readers to understand faster if a representative ground photograph showing the sampling points marked with the bamboo stakes can also be included in Figure 1.
🡪 Sampling point marked with bamboo stakes is in S1 Fig now.
Regarding collection of water sample:
L136: It was described that water "...were collected into new 500 ml plastic bottles. Suggestion for clarity and reproducibility of method by other researchers: state the approximate volume of water collected in this study.
🡪 Approximately volume is in lines 154-155.
i) How was the water collected into the 500 ml plastic bottles?
ii) Was the plastic bottle directly immersed into the water body? OR A water dipper/ladle was used to ladle the water into the plastic bottles?
iii) Were the plastic bottles pre-sterilized type?
🡪 The information is provided in paragraph lines 147-166 .
Regarding transport of water & soil samples:
i) How were the samples transported after sampling was completed?
ii) How soon were the samples transported to the lab and processed?
🡪 The information is provided in paragraph lines 147-166.
Regarding samples processing & handling?
i) How were the water and soil samples processed in the lab prior to plating and molecular analysis?
🡪 The information is provided in paragraph lines 147-166.
ii) How were the samples stored before and after processing (e.g. storage temperature & etc.)

🡪 The information is provided in paragraph lines 147-166.
Regarding Quantitative Culture for B. pseudomallei:
L166-168: "...500 uL of water sample... was directly plated..."
i) Was the 500 uL water sample pipetted all at once onto the agar plates? OR The 500 uL sample was divided into 2-3 portions, and pipetted onto the agar portion by portion, allowing the agar to dry before pipetting the next portion? This question is asked because an agar plate prepared using the commonly used petri dish (Diameter 90mm, Depth 15mm) can typically absorbed approximately 200 uL of plating liquid at the maximum at one time. To safely plate a volume of 500 uL all at once, it is assumed that larger-sized petri dishes were used in the current study. If this was the case, what was the size of the petri dishes used to plate the 500 uL water sample?
🡪 The used 200 ul for plating out. The information is corrected in line 194.
L169: "...other bacterial colonies were counted..."
i) What species or genus constitute the "other bacteria"?
ii) How were colonies of the "other bacteria" differentiated from B. pseudomallei colonies on the agar plate?

🡪 The information is provided in lines 193-203.

**Results**
Reviewer #1: See below

Reviewer #2: Results are clearly presented, with the exception of confusion being created lines 223-227 referring to studies that were not described in methods.

🡪 The method is described in lines 193-203.
Figures are clear and appropriate, with one exception (Figure 4 requires an axis label for the horizontal axis); narratives describing figures are appropriate and comprehensive

🡪 Labels are provided in the figure 4.

Reviewer #3: In general, the results presented matched the planned analysis techniques and statistical methods. Following are areas where questions and comments surfaced during review of the manuscript:
L225: Is the word "uncountable" in this context meant that there were no bacteria growing on the plate or there were too many bacteria/microorganisms growing on the plate that it was not possible to count the colonies?
🡪 We changed to “undetectable” line 261.
Regarding the 40 human melioidosis case data obtained from the hospitals:
If the data was available, it is suggested that the basic demographics of the 40 cases are summarized in a table and included in the result section, information such as patients' age, sex, and occupation. Justification for inclusion: in the absence of this information in the current manuscript, it can only be assumed that all the 40 human cases were rice farmers, which the text in line 233-234 (The last cluster observed after the harvesting season of the second rice crop) seemed to imply.
🡪 We added a S3 Table describing the basic demographics of patients in supplemental data.
FIGURE LEGEND:
L494: The dashed bars (showing the monthly average weather variables) took a bit of time to spot in the graphs (due to multiple data being squeezed into the figure).
🡪 As your suggestion, now we present only the main data in the figure 2, the other data is moved to supplemental S5 figure. The graphs in the figure 2 look greater now.
Line 506: Regarding the phrase "right and left water sample", it is suggested that the description is amended to "...are water samples collected on the right-hand path and left-hand path of each sampling point, respectively", consistent with the text used in the Method section (Line 135)
🡪 Corrected in line 488-489.
FIGURES:
Figure 1 (b): It is suggested that a correctly orientated compass point is included in this figure.
🡪 Compass is included.
Figure 2:
i) Since only ambient temperature demonstrated significant positive correlation with B. pseudomallei positive culture rate, Figure 2 perhaps can be simplified to showcase the ambient temperature data. The rest of the data (humidity, windspeed and precipitation) could be included as Supplementary.
🡪 Revised as your suggestion.
ii) Is "1 melioidosis case" equaled to one confirmed case, and "2 melioidosis case" equaled to two confirmed cases?
🡪 Yes, mean the circle sizes are proportion to the number of cases admitted to hospital on a that day, describing in line 501-502.
Figure 4:
i) It is suggested that the annotation "Sampling points" on the horizontal axis, and "Sampling date" on the vertical axis are included.
🡪 Revised as your suggestion
ii) Hopefully a high resolution of this figure is available. Also, it is suggested that a lighter shade of blue is used to denote the sites that were positive for B. pseudomallei culture. At the current resolution and color combinations, it was challenging to see which sampling sites had which STs.

🡪 In our current resolution, the figure looks very clearly. I understand that the reviewers are received a low resolution of figures from PLOS journals during reviewing process.

**Conclusions**
Reviewer #1: See below

Reviewer #2: Conclusions are supported by the data; would request limitation be added related to not measuring water temperature at the sampling site at the time of sample collections. Public health relevance is addressed, and study links effectively to clinical findings that were described

🡪 This information is added in line 369-370.

Reviewer #3: The conclusions are generally supported by the results presented. The limitations were also mentioned, in particular the awareness that melioidosis cases nearby the study site were not forthcoming. However, it is probably a little less accurate to deduce that melioidosis cases were under-reported in year 2018 at the Thanh Hoa province just because more cases were detected in the subsequent years.
🡪 As requested by editors, we added number of cases in Thanh Hoa province in 2019 and 2020 (lines 375-378), to demonstrate that this is endemic area of melioidosis.
An aspect that was not discussed was why paddy field was selected as the preferred study site, as compared to other type of aquatic environments.
🡪 We explain the reason to select this study site in lines 135-146.
Following are some comments and questions on the Discussion section:

L270: For microorganisms, the term culturability is typically used, e.g. Viable but non-culturable
🡪 Revised in line 309.
Line 306: Sounds contradicting. The sentence "groundwater level actually followed the rise of melioidosis cases" is not clear in the context of this paragraph.
🡪 This paragraph is deleted, as suggestion from reviewer #1.
Line 302-Line 315: Have difficulty relating the discussion on the effect of ground water level on the bacterial load since the current study did not look at the effect of ground water level.
🡪 This paragraph is deleted, as suggestion from reviewer #1.
Line 352: Was it "irritations" or "irrigations"?

🡪 Revised in line 373.

**Editorial and Data Presentation Modifications?**
Reviewer #1: See below

Reviewer #2: The following are suggested edits and revisions:
Line 119, correct “spring” to “autumn” prior to “from September to November”

🡪 Revised in line 127.
Line 126, consider word change from “water” to “paddy fields” prior to “always remain flooded”

🡪 Revised in lines 133-134.
Recommend reorganizing paragraph lines 115-131 to generally describe the region and rice harvest, followed by description of the pilot study (for example, move the first sentence of the paragraph lines 115-116 to line 126 prior to “At each sampling site”, and open the paragraph instead with “The study site lies in north-central Vietnam.”)

🡪 Revised as your suggestion, now in lines 124-134.
Line 130, consider changing “was completely run out of” to “was drained of”

🡪 Revised
Line 131, consider adding phrase “Based on the pilot study results,” before “this sampling site…”

🡪 Revised in line 145.
Consider moving the sentence in lines 104-141 to Line 137 before “At each visit” for better continuity

🡪 Now, the paragraph in lines 147-166 is revised.
Line 141, consider changing “It” to “Sampling”

🡪 Revised
Line 178, consider changing “raising on” to “trainings on”

🡪 We keep “raising on” in line 211.
Paragraph lines 223-227 – this paragraph does not make sense – what “other bacteria” is being referred to? Need to include reference to this in the Materials and Methods, describing culture of “other bacteria”; also, were these bacteria identified? This is very important information to include given that these bacteria were in competition with B pseudomallei in the environment and may have influenced the ability to culture quantitatively

🡪 Method to quantify other bacteria is added in lines 193-203.
The first sentence of paragraph lines 235-239 is a bit confusing on first read; consider re-wording for greater clarity.

🡪 Revised in lines 271-278.
Line 242, consider deleting “requires a complex of” as this confusing wording; consider instead replacing with the word “involves”

🡪 Revised in line 281.
Lines 250-253 – need a reference for this sentence
🡪 a reference is added in line 291.
Figure 4 – need to have label for horizontal/x-axis

🡪 Revised

Reviewer #3: Revision of this manuscript falls in between Minor and Medium Revision.

**Summary and General Comments**

Reviewer #1: PNTD-D-25-00046
"Seasonal change of Burkholderia pseudomallei in paddy field water strongly correlates
with ambient temperature: A study in north-central Vietnam."
Vu, A.T.N., et al.
This paper evaluates the presence of Burkholderia pseudomallei in paddy fields in Vietnam and the impact seasonal changes are having in the isolation of the bacteria in the soil. The data indicates that changes in temperature have a direct impact in the number of bacteria recovered from the sampling sites, which is an important finding for endemic areas. However, the manuscript requires significant editing and interpretation of the data requires further analysis. The authors should strongly consider the following points to improve the paper:
Major points:
1. Unfortunately, the manuscript requires significant attention to the grammar and additional editing of the manuscript is required. Normally I do not consider that an issue, but this manuscript requires attention, so the data presented can be highlighter. Some examples are provided below.

🡪 Based on reviewer’s comments and suggestions, we edited the manuscript significantly.
2. Abstract lane 43, is prevalence what is impacted by changes in ambient temperature? Although prevalence is defined as the number of subjects either at a specific point in or over a specified period, the changes in temperature are only affecting the threshold of bacterial recovery because the microorganism is already prevalent in the soil.

🡪 Thank you for a great comment. We changed to “occurrence” in this line and throughout the manuscript.
3. Labeling of the panels and description in the text needs to be improved. For example, in lanes 138-140, the panels A through e need to be depicted in the text.

🡪 Yes, all are revised.
4. Fig 2, lanes 141-143 and discussion, it is evident that case clusters 1 and 3 do not match case cluster 2 with respect to average temperature and precipitation. This is an important point of discussion because you might expect, based on the overall premise, that increase in temperature also produce an increase in bacterial recovery and perhaps cases. However, the data does not support this trend or demonstrate increase in cases in specific time of the year of with environmental conditions. Has this phenomenon observed elsewhere in southeast Asia, in Australia?

🡪 Based on the small number of cases observed in 2018, we could not provide a detailed discussion or draw conclusions about this phenomenon. A time series analysis, including cases from multiple years, will be needed to clarify or explain this in the future.
5. Also in Fig 2, the positive culture rate seems to be restricted to two monthly periods, April-June and July to October, which correlates with increase in temperature but not with increase precipitation. Any thoughts about these discrepancies?

🡪 Yes, we found a correlation between the positive culture rate and temperature, and we focused on the discussions based on the observation.
6. Figure 3 is hard to interpret with clear explanation in the text. What is the meaning of half positive/half negative squares? Further, the color dots describing the ST types are not easily visible and the blue background interferes with visualization. Further, what is the meaning of detecting a ST type in half of the square and not in the other, in squares that are all positive?

🡪 The legend of Figure 4 is revised. In our current resolution, the figure looks very clearly. I understand that the reviewers are received a low resolution of figures from PLOS journals during reviewing process.
7. Lanes 264-265, do the ambient temperature contribute to the growth and persistence? I would argue that ambient temperature contributes to the increase isolation of the bacteria in areas of known prevalence of the pathogen

🡪 We think both growth and persistence.
8. Finally, the discussion is extensive (5.5 pages) and in areas quite confusing. It is highly recommended to streamline the discussion and reduce the length, highlighting the results and comparing with other studies.

🡪 We deleted some sentences and paragraphs not related to the findings.
9. Lanes 343-344, it is the presence of ST 1994 paddy-specific, or it is a new subtype of concern in Vietnam?.
🡪 This is new ST. Further study on whole genome sequencing is needed to address your questions.
Minor points:
1. Abstract, lane 29, “…focused on water samples at a…”

🡪 corrected in line 29.
2. Lanes 31-32, “B. pseudomallei real-time PCR…gene and also for B. pseudomallei…”.

🡪 corrected in lines 31-32.
3. Lane 34, “…quantitative culture method directly…”

🡪 corrected in line 34.
4. Lane 56 “…during the summer…”

🡪 corrected in line 56.
5. Lane 92, what is “cloud cover”?

🡪 this is a meteorological term that we referred from the study by Kaestli et al. 2016
6. Lane 119, do you mean: “…August, and autumn…”

🡪 corrected in line 127.
7. Lane 124, the following “particularly rice production” is repetitive and the sentence needs to be revised.

🡪 we revised the paragraph in lines 130-132.
8. Lane 164 “…B. pseudomallei RT-PCR…”

🡪 corrected the paragraph in lines 163-165.
9. Lane 184 “(Fig 1A)”

🡪 corrected
10. Lane 212, what figure panel? Likewise in lane 219 and 222.

🡪 Revised
11. Lanes 242-244, please revise sentence, particularly in the concept of “complex of multiple factors”

🡪 revised
12. Lanes 256-257, “…rainfalls start typically in the middle of…”

🡪 the sentence was revised in lines 293-295.
13. Lane 271 “…In the environment mimicking…”

🡪 revised in line 310.
14. Lane 280, sentence is redundant: “…an increase to the optimal growth temperature…”

🡪 revised in 319
15. Lanes 313-315, this sentence is speculative, and data does not support this statement.

🡪 this sentence was deleted
16. References have to be formatted accordingly.

🡪 Yes

Reviewer #2: A strong study overall in my opinion, with a novel approach that is quite relevant to the epidemiology of melioidosis.
I do have one limitation that I would request be added, with relevant discussion:
🡪 Limitation of not measuring water temperatures is added in line 369-370.
Were water temperatures also measured? I am curious whether the drop in the culture rate noted in the August sample may have been due to decreased water temperature secondary to the heavy rains associated with TD1, given that average temperature remained constant from July to October, as an alternative explanation vs dilution mentioned in the manuscript.

🡪 We prefer dilution. Because the tropical rainfalls are quite heavy.

Reviewer #3: In summary, this study and its findings have sufficient merits to be published. Many researchers in the B. pseudomallei endemic regions will be interested to read up on it. The main area needing improvement is the method section. It would be of great service to other researchers if this section, in particular the sampling and B. pseudomallei isolation and culture section is meticulously described in detail.
🡪 We revised as your comments and suggestions.
Final recommendation: It may help improve readability further if English language assistance was obtained.

🡪 Yes, we pay our attentions to revise the manuscript.
