# Supplementary material for: Seasonal change of Burkholderia pseudomallei in paddy field water strongly correlates with ambient temperature: A study in north-central Vietnam
Source: PLoS Negl Trop Dis. 2025 Jul 30;19(7):e0013322. doi: 10.1371/journal.pntd.0013322 (PMC12321097; doi:10.1371/journal.pntd.0013322)
Supplement: Attachment — Submitted filename: Response_to_Reviewers_auresp_2.docx [file pntd.0013322.s011.docx]

Dear reviewers,

Thank you very much for your carefully comments on our final manuscript. In this letter, we provide our responses to each of your comments below.

Best regards,

Trung

**Reviewer's Responses to Questions**

**Methods**

Reviewer #2: Objectives are clear, study design appropriate for the stated objectives. Sample size sufficient, and correct statistical analyses. All issues identified by reviewers appear to have been adequately addressed.

In looking at Figure 1A, consider adding the word "confirmed" for the figure text, as the manuscript refers to only confirmed cases (very minor issue)

🡪 The “culture-confirmed” word was added. Additionally, we mentioned “melioidosis” to patients (line 497) to clarify the subject.

**Editorial and Data Presentation Modifications?**

Reviewer #2: Line 90 - I would suggest one minor word change, from "retains" to "replenishes"

🡪 We change the word as your suggestion.

Line 124-125 - Would reword for clarity- I think a word or two is missing (e.g. located between two climate classifications/types

🡪 We added “types” as your suggestion.

**Summary and General Comments**

Reviewer #1: PNTD-D-25-00046R1
This is a significantly improved paper evaluating the presence of Burkholderia pseudomallei in paddy fields in Vietnam and the impact seasonal changes are having in the isolation of the bacteria in the soil. The authors were responsive to the reviewers’ requests, and I only have two minor points to correct:

Minor points:
1. Within the results section, figure 1 panels C, D and E should be discussed and not just described in the figure legend.

🡪 We discussed and cited these figure panels from line 298 to 303.

2. Similarly, Figure 4 should be better described in the results section and not just in the figure legend.

🡪 We described the Figure 4 in the result section, from line 271 to 277.
